# THE LONGEVITY ASSOCIATED ALLELE OF FOXO3 PROTECTS AGAINST TELOMERE ATTRITION DURING AGING

**DOI:** 10.1093/geroni/igz038.374

**Published:** 2019-11-08

**Authors:** Richard Allsopp, Philip Davy, Craig Willcox, Randi Chen, Michio Shimabukuro, Immaculata de Vivo, Bradley J Willcox

**Affiliations:** 1 University of Hawaii, Honolulu, Hawaii, United States; 2 Okinawa International University, Ginowan, Okinawa, Japan; 3 PHREI, Honolulu, Hawaii, United States; 4 Fukushima Medical University, Fukushima, Okinawa, Japan; 5 Harvard T. H. Chan School of Public Health, Boston, Massachusetts, United States; 6 Kuakini Medical Center, Honolulu, Hawaii, United States

## Abstract

Telomere attrition in proliferative tissues is a hallmark feature of human aging. To date, the genetic influence on the rate of telomere attrition is poorly understood. Previously we discovered a variant of the FOXO3 gene that is strongly associated with human longevity, an observation that has been now reproduced in over a dozen independent studies. In the present study, we sought to assess the effect of the longevity associated variant of FOXO3 (rs2802292 - G allele) on the rate of telomere attrition during aging. The results from a cohort of Okinawan-Japanese (N=121), ranging in age from 25 – 94 years, demonstrates carriers of 1 or 2 copies of the longevity-associated G allele of FOXO3 showed markedly reduced rates of telomere loss in peripheral blood leucocytes as compared to carriers of the more common FOXO3 variant (TT – common genotype, m= -33bp/year, P=0.008). Interestingly, telomere shortening was not observed as a function of age for G allele carriers (m= -2bp/year, P>0.1). In an independent study of women (N=6,565) from the Nurses’ Health Study cohort, ranging in age from 40 to 70 years, a similar observation was found. Notably, carriers of the TT or GT FOXO3 genotype showed a significant decline in telomere length with age (m= -15.5 bp/year, P0.1). These results mark the first validated longevity gene variant showing an association with negligible loss of telomere length with age in humans.

